# Soft Tissue Retraction Maneuver in Cone Beam Computed Tomography Prior to Crown-Lengthening Procedure—A Technical Note

**DOI:** 10.3390/jcm13133668

**Published:** 2024-06-24

**Authors:** Paulina Adamska, Marcin Stasiak, Wojciech Dąbrowski, Dorota Pylińska-Dąbrowska, Łukasz Jan Adamski, Adam Zedler, Ewa Kozłowska, Michał Studniarek

**Affiliations:** 1 Division of Oral Surgery, Faculty of Medicine, Medical University of Gdańsk, 7 Dębinki Street, 80-211 Gdańsk, Poland; adam.zedler@gumed.edu.pl; 2 Division of Orthodontics, Faculty of Medicine, Medical University of Gdańsk, 42c Aleja Zwycięstwa, 80-210 Gdańsk, Poland; marcin.stasiak@gumed.edu.pl; 3 Department of Dental Prosthetics, Faculty of Medicine, Medical University of Gdańsk, 18 Orzeszkowej Street, 80-204 Gdańsk, Poland; wojciech.dabrowski@gumed.edu.pl (W.D.); dorota.pylinska-dabrowska@gumed.edu.pl (D.P.-D.); 4 Private Dental Practice Łukasz Adamski, 3B Stawiska, 83-431 Stary Bukowiec, Poland; lukasz.adamski@gumed.edu.pl; 5 Institute of Manufacturing and Materials Technology, Faculty of Mechanical Engineering and Ship Technology, Gdańsk University of Technology, 11/12 Gabriela Narutowicza Street, 80-233 Gdańsk, Poland; ewa.kozlowska@pg.edu.pl; 6 Department of Radiology, Faculty of Medicine, Medical University of Gdańsk, 17 Smoluchowskiego Street, 80-214 Gdańsk, Poland; michal.studniarek@gumed.edu.pl

**Keywords:** cone beam computed tomography, crown lengthening, gingiva, gingivoplasty, osteoplasty, periodontium, tooth cervix, tooth crown, tooth socket

## Abstract

**Background:** An accurate determination of the biological width and the relationship of the cemento-enamel junction with the border of the alveolar bone is crucial during a clinical crown-lengthening (CCL) procedure. The aim of this study was to present a technical note about the retraction techniques in cone beam computed tomography (CBCT) prior to CCL, highlighting the significant enhancement in procedural accuracy and predictability that these techniques offer. **Methods:** Clinical and radiological examinations should be performed before a CCL procedure. It is necessary to determine the length of the tooth crowns, the periodontal pockets’ depth, and the phenotype of the gingiva. The ideal CBCT examination should be performed with soft tissue retraction. This can be achieved using retractors or cotton rolls. **Results:** Retraction of the lips, cheeks, and tongue allows one to assess the marginal gingiva, the cemento-enamel junction, and the alveolar bone. A detailed plan of the CCL procedure, which involves retraction, ensures both the aesthetic appeal and the achievement of a newly defined gingival zenith, enhancing the overall visual harmony. **Conclusions:** Compared with conventional radiographic imaging, the soft tissue retraction maneuver in CBCT prior to CCL surgery offers an effective approach to the evaluation and diagnosis of soft and hard tissue. This is because of the detailed planning of the aesthetic CCL procedure. Such an approach leads to superior aesthetic outcomes in dentistry, contributing to the advancement of aesthetic dentistry through a harmonious blend of art and science.

## 1. Introduction

It is very important to maintain a harmonious smile, perfect aesthetics of red and white, and the golden ratio. The golden ratio, i.e., the phi number (φ), has been known since antiquity and was established around the sixth century BC. According to the golden ratio paradigm, the ideal ratio is approximately 1:1.618. The first implementation of the golden ratio into dentistry was conducted by Lombardi. He noted that the central and lateral incisors may have a constant size relationship. This issue was expanded by Levin. He estimated that the width of the central incisor (1.618) is in golden proportion to the width of the lateral incisor (1.0). The width of the lateral incisor is related to the width of the canine (0.618). These measurements are taken en face of the smile. Another important analysis is the RED (recurring esthetic dental) assessment. Ward estimated that when the patient looks straight ahead, each subsequent tooth becomes smaller and smaller, evaluating the face from a frontal perspective. This assessment underlines the importance of gradual diminution in tooth size from the central incisors to the canines, adhering to a natural, aesthetically pleasing progression [1,2,3,4,5].

Clinical crown lengthening (CCL) is one of the most common procedures in periodontal surgery, addressing aesthetic concerns, inflammatory problems related to exceeding the biological width (aesthetic reasons), or reproducing the ‘ferrule effect’ (functional CCL; a ‘ferrule effect’ is a situation when there is a 1.5–2 mm band of dentin stroma around the tooth above the gingiva, which ensures the tooth has adequate resistance to fracture). Indications include subgingival caries, crown or root fractures, passive eruption, cervical resorption, and restoring the correct biological width. CCL is performed concomitantly for aesthetic reasons when teeth crowns are too short or as one of the methods of treating a gummy smile. The procedure aims to adjust the gingiva line and, if necessary, the bone level to expose more of the tooth’s crown, or to create space for restorative materials without violating the biological width—a critical factor in preventing periodontal diseases and ensuring the longevity of dental restorations, especially in the anterior sections of the maxilla [6]. In orthodontic treatment, incisors’ intrusion moves the dental gingival complex apically, providing improvement of the gingival margin’s level. Therefore, it is reasonable to lengthen the crowns at the end of the active orthodontic treatment or in the retention phase [7]. In instances of hereditary gingival fibromatosis, it is imperative that CCL procedures be executed prior to the initiation of orthodontic therapy, to facilitate the subsequent placement of orthodontic appliances. During orthodontic treatment, further CCL should also be considered due to frequent recurrences [8].

CCL procedures are divided into gingivoplasty and gingivoplasty with osteoplasty (or gingivo-osteoplasty). Gingivoplasty is usually performed to reshape healthy gingiva tissue. Gingivoplasty with osteoplasty involves the simultaneous reduction of gingiva and bone excess (bone modeling horizontally—osteoplasty and vertically—ostectomy). Gingivoplasty is performed if the distance from the cemento-enamel junction (CEJ) to the edge of the alveolar bone is correct. It can be performed with a scalpel, electrocautery, or laser. If the biological width is not maintained, in addition to removing excess gingiva, bone reduction is performed. Osteoplasty with ostectomy is performed using rotary and ultrasonic tools, as well as manual chisels and files. The extent of bone reduction depends on the phenotype of the patient’s gingiva. The gingival phenotype is assessed using a periodontal probe. The thin phenotype is characterized by a gingival thickness of less than or equal to 1 mm and the periodontal probe showing through the gingiva when the probe is inserted into the gingival pocket. A thick phenotype is described when the gingival thickness is greater than 1 mm and the probe is not visible through the gingiva when inserted into the pocket. In patients with a thin phenotype, the distance between the cemento-enamel junction and the bone edge should be 2.5 mm, whereas in patients with a thick gingival phenotype, it should be 3.5 mm. Moreover, if the gingiva is 1 mm thick, osteoplasty should be performed within 3 mm [6,9,10,11,12].

The free gingival margin requires about 3 months to establish its final vertical position [13]. This is the minimum healing time of the free gingival margin. The healing time depends on anatomical, physiological, and biological factors, i.e., the gingiva phenotype, the initial position of the bone in relation to the CEJ (biological width), the amount of reduced bone and the modeling of the alveolar bone, the adaptation of the mucoperiosteal flap after the procedure, and the traumatic nature of the procedure and clinician experience. In aesthetically demanding parts of the oral cavity, this period may last half a year. Therefore, only temporary restoration should be performed after CCL surgery. A final prosthetic treatment should be postponed until the obtainment of the stability of soft tissue anatomy [11,14,15,16].

Cone beam computed tomography (CBCT) is a basic examination used in dentistry in situations where conventional X-rays do not allow one to obtain sufficient accuracy for diagnostic purposes [17,18,19,20,21,22,23,24,25,26,27,28,29]. CBCT is primarily used to assess pathology in hard tissues. The use of additional techniques can also ensure diagnosis and treatment planning in the soft tissue area. CBCT enables very good resolution of the examination with a relatively low dose compared to computed tomography (CT) [23,24]. The ‘puffed cheek’ maneuver is described as a method supporting CT diagnostics of lesions in the oral cavity, oropharynx, hypopharynx, and nasopharynx. By inflating the oral cavity and mouth vestibules with air while the patient’s lips remain sealed during the scan, this technique improves soft tissue lesion detectability and delineation [24,25,26,27,28,29]. Other techniques include the modified Valsalva and phonation maneuvers. In Valsalva, expiration is performed against the resistance of pursed lips or nose. The patient holds their breath for 10 s. During the phonation maneuver, sustained pronunciation of the letter ‘e’ for a similar duration may aid in assessing soft tissue structures within the scanned area [25]. Determining the dimensions of periodontal tissues is important for planning a surgical procedure, for example, CCL [30,31].

The aim of this study was to present a technical note about the retraction techniques in cone beam computed tomography prior to CCL, highlighting the significant enhancement in procedural accuracy and predictability that these techniques offer.

## 2. Materials and Methods

**Figure 1:**
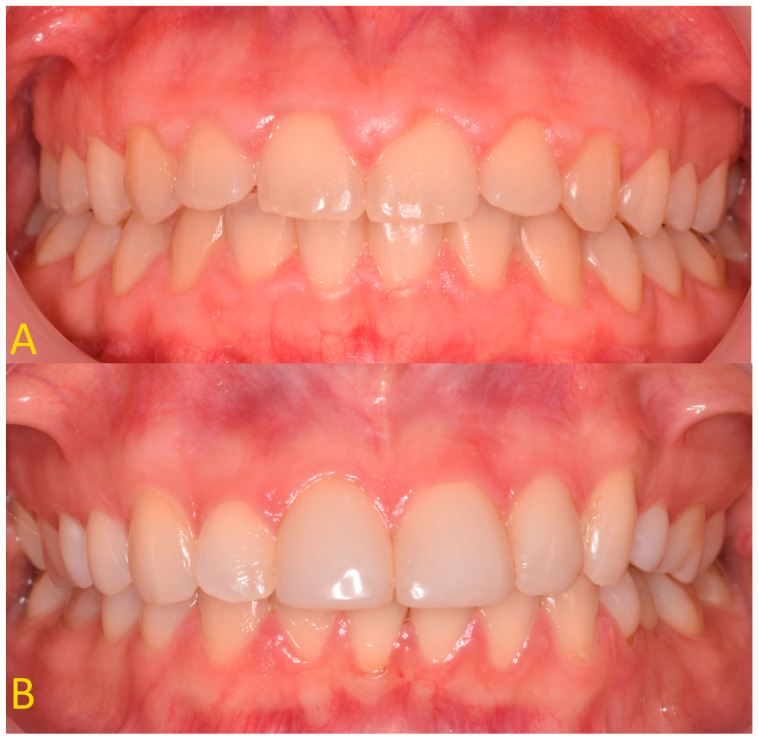
(**A**) Patient with shortened clinical crowns in the maxilla and mandible—thick gingival phenotype; (**B**) patient with shortened clinical crowns around teeth 13, 12, and 21 (according to the Viohl classification = FDI (fr. *Fédération Dentaire Internationale*))—thin gingival phenotype.

Both a clinical and a radiological examination should be performed before prosthetic treatment. It is necessary to take photographic images of the patient’s face at rest, smiling slightly, smiling as much as possible, and intraorally with retractors. Moreover, the assessment according to the pupillary line is very important for the smile aesthetic. Extraoral photos are very important because they allow an aesthetic analysis to be performed. Based on the analysis of facial thirds and smile symmetry, initial smile visualization is performed using digital tools. Patients with short clinical crowns, which are inconsistent with the principles of the golden ratio, and an excessive biological width are eligible for an aesthetic CCL procedure. When the ‘ferrule effect’ cannot be achieved, patients are eligible for functional CCL surgery. The most important information from the clinical examination is as follows: the length of the crowns, the periodontal pockets’ depth, and the phenotype of the gingiva (Figure 1). The gingival phenotype is assessed using a periodontal probe as previously described. 

**Figure 2:**
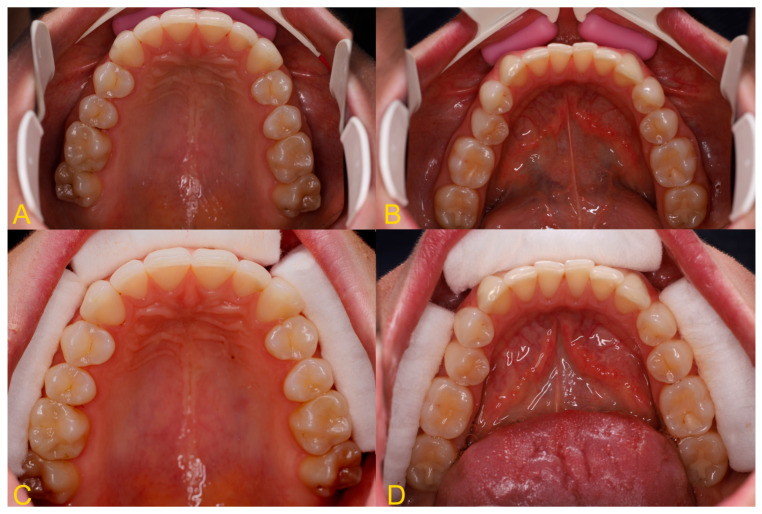
Intraoral photography—soft tissue retraction using (**A**,**B**) dental retractor and (**C**,**D**) dental cotton rolls.

**Figure 3:**
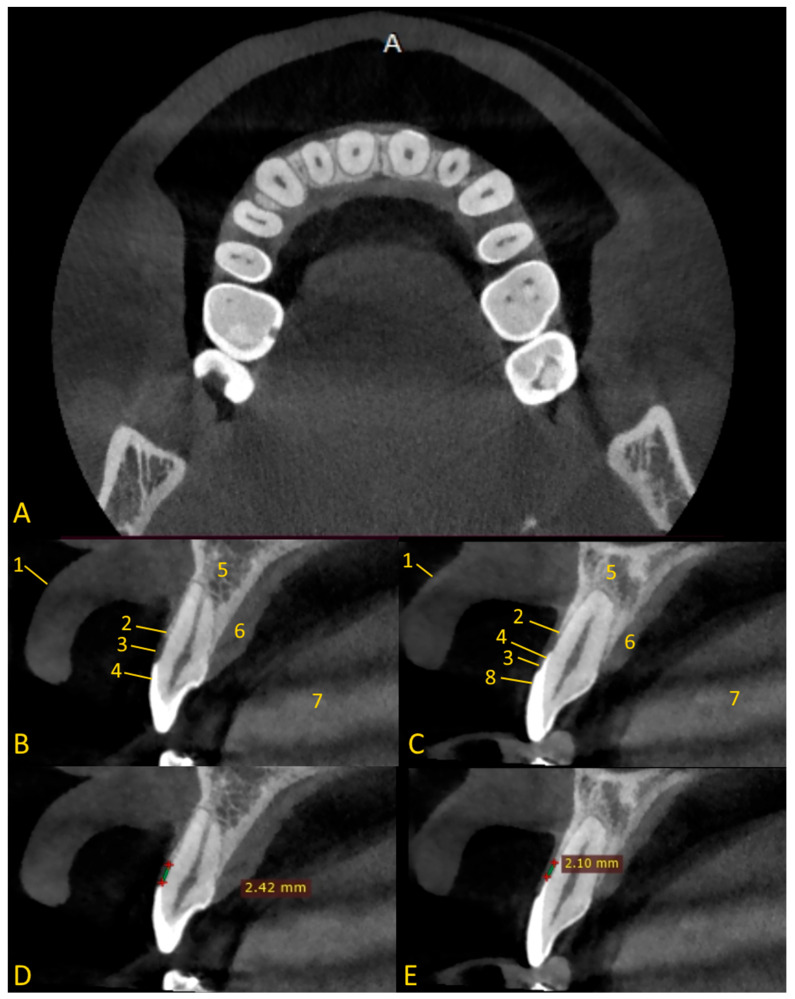
Soft tissue retraction maneuver in cone beam computed tomography. Cases of soft tissue retraction, after viewing the CBCT images, can be assessed to clearly distinguish between the gingiva and the soft tissue on the palate. In cases where soft tissue retraction is not available, we are unable to clearly differentiate between the lip and gingiva: (**A**) axial view—lip retraction using cotton rolls; (**B**) cross-sectional view, tooth 12 without composite restoration—enamel is under gingiva; (**C**) cross-sectional view, tooth 21 with composite veneer—enamel is under gingiva, the veneer does not cover all of the enamel on the labial side; (**D**) cross-sectional view, tooth 12—distance between the CEJ and the edge of the alveolar bone; (**E**) tooth 21—distance between the CEJ and the edge of the alveolar bone. 1—lip; 2—bone edge margin; 3—gingiva; 4—enamel; 5—alveolar bone; 6—soft tissue of palate; 7—tongue; 8—composite veneer.

**Figure 4:**
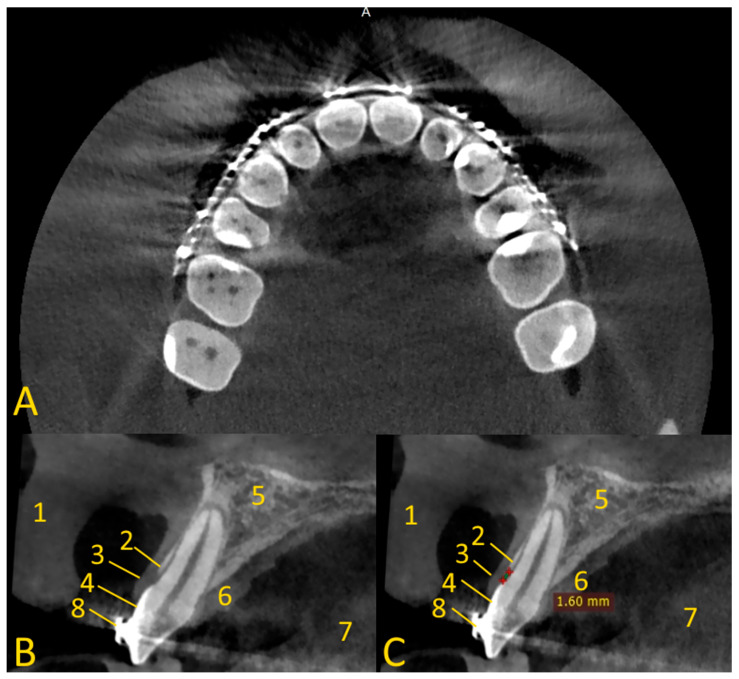
Soft tissue retraction maneuver in cone beam computed tomography in patient with orthodontic appliance (numerous artifacts are visible). Cases of soft tissue retraction, after viewing the CBCT images, can be assessed to clearly distinguish between the gingiva and the soft tissue on the palate. In cases where soft tissue retraction is not available, we are unable to clearly differentiate between the lip and gingiva: (**A**) axial view—lip retraction using cotton rolls; (**B**) cross-sectional view, tooth 12—enamel is under gingiva; (**C**) distance between the CEJ and the edge of the alveolar bone is very short. 1—lip; 2—bone edge margin; 3—gingiva; 4—enamel; 5—alveolar bone; 6—soft tissue of palate; 7—tongue; 8—metal orthodontic bracket.

**Figure 5:**
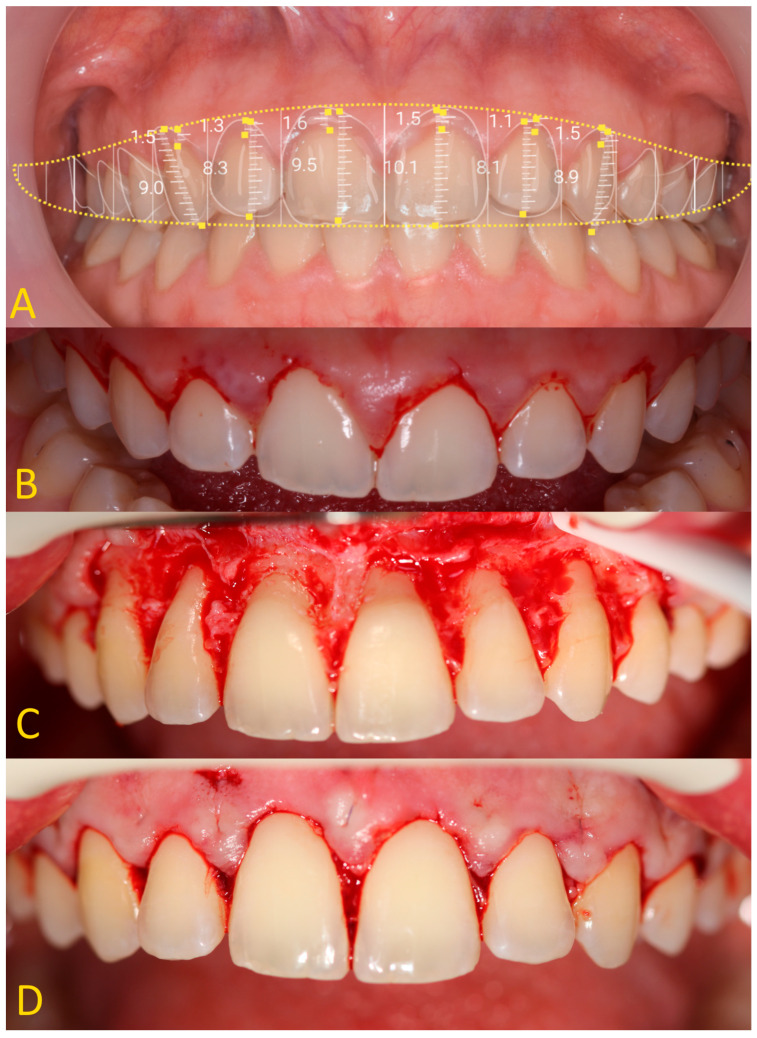
Crown-lengthening procedure—intraoral photography: (**A**) aesthetic analysis, gingivectomy, and ostectomy measurements; (**B**) after gingivectomy; (**C**) after ostectomy; (**D**) after wound suture.

The soft tissue retraction technique involves performing a CBCT examination under special conditions. Before performing the examination, cotton rolls or a retractor are inserted between the upper and lower lips and cheeks, and the alveolar process of the maxilla or the alveolar part of the mandible (Figure 2). The patient must stand still, the head should be fixed in the positioner, and the teeth must bite the bite bar. The examination must be performed by a qualified physician with radiation protection training or by a radiology technician. This provides repeatability and protection against motion artifacts that would affect the quality of the examination. During the examination, the patient’s tongue must rest on the floor of the oral cavity (Figure 3 and Figure 4). Moreover, a gauze pad can be placed between the palate and the tongue. The imaging conditions adopted in this study were 110 kV, 5 mA, voxel size of 0.2 mm, field of view (FOV) of 7 × 10 cm, and CTDIvol of 4.62 mGy (best quality protocol). With modern CBCT machines, the radiation dose in CBCT of the anterior maxilla may be lower than the radiation dose level in a panoramic CT scan, with a much greater scope of information that can be obtained. In such a setting, the popular bite-wing radiographs may have a higher dose than CBCT and do not provide information about soft tissues. ‘Best quality protocol’ was used due to the higher resolution of CBCT and the smallest possible distances between scans, thanks to which the obtained images are the most accurate. In this study, the scans were performed using MyRay Hyperion X9, Stern Weber, Switzerland (resolution up to 68 μm). Computed tomography scans were evaluated in iRYS software (MyRay Hyperion X9, Stern Weber, Switzerland). In the case of a patient wearing a fixed appliance (Figure 4) or prosthetic restorations, numerous artifacts are visible. Based on clinical and radiological examination, gingivoplasty and osteoplasty values are precisely determined. The final digital smile design can be made using basic graphic programs or by using special programs, e.g., SMILE CLOUD (Timișoara, Romania; Figure 5A).

The surgical procedure is performed under local anesthesia (4% articaine hydrochloride containing 1:100,000 epinephrine; e.g., Molteni Dental s.r.l., Scandicci, Florence, Italy). If a patient has fillings, veneers, or prosthetic crowns, the quality of their work should be assessed. If the composite fillings or veneers are incorrectly shaped, they should be corrected before or during the surgical procedure. If crowns do not fit well, they should be replaced with temporary crowns. The filling/veneer/crown must end at the CEJ level and must be perfectly fitted, smooth, and freely flow into the root cementum. 

In the first stage of the CCL procedure, gingivoplasty is performed (Figure 5). Excess gingiva is removed according to the planned values. A periodontal probe (e.g., CP 15 North Caroline periodontal probe Helmut Zepf Medizintechnik GmbH, Seitingen-Oberflacht, Germany), prosthetic thickness gauge (e.g., Orimed, Osiec, Poland), or Castroviejo implant measuring device (e.g., Orimed, Osiec, Poland) can be used to measure the extent of the tissue removed. Another method of determining the scope of the procedure may be to use a surgical guide. The template has two boundaries—the gingiva and bone removal range. In addition to CBCT, an intraoral scan (IOS) is needed to create the surgical guide [32]. Gingiva can be removed with a scalpel 15 c (Swann Morton, Dorset, FA, UK), electrocautery (e.g., Surtron 160. Eres Medical, Tomaszowice, Poland), or laser (e.g., Fotona LightWalker AT-S, Irving, TX, USA). 

If there is not an adequate distance between the CEJ and the alveolar bone margin, osteoplasty with ostectomy should be performed. Vertical bone reduction can be performed using a closed method using special ultrasonic tips (without the elevation of the mucoperiosteal flap, e.g., Piezo Ultrasonic Surgery Osteotomy Bone Surgery Tips Set, DoWell, Rancho Cucamonga, CA, US) or an open method. In the case of the open technique, a full-thickness mucoperiosteal flap should be detached only to the mucogingival line (MGL). This reduces the risk of postoperative swelling. Elevation of the flap above the MGL increases the reaction of the soft tissues and causes edema. The osteoplasty procedure is performed both vertically and horizontally. First, the bone is reduced in thickness, horizontally. This can be performed using a ball drill (e.g., round bur *Meisinger, Hager & Meisinger GmbH, Neuss, Germany*) on a handpiece or accelerating contra-angle (e.g., W&H Dentalwerk Bürmoos GmbH, Bürmoos, Austria). In the same way, the bone is removed vertically. To protect the root, the last layer of bone should be removed using manual chisels and files (e.g., no nr 3/8mm M 649/3-7.HL8 Hirschfeld perio chisel, Scharf Instruments, Nürnberg, Germany). An ostectomy should be performed depending on the patient’s biotype: thick—3.5 mm from the CEJ line, normal—3 mm, and thin—2.5 mm. During the procedure, the flap should be moistened with 0.9% sodium chloride. The new zenith of the gingiva and bone must coincide with the zenith of the tooth. The wound is sewn with thin monofilament non-resorbable 6-0 sutures. In the anterior section, the best adaptation of the mucoperiosteal flap is provided by vertical mattress sutures. The interdental papillae are high and the vertical mattress sutures tighten the tissues well. In the lateral section, the interdental papillae are low, so single sutures should be used. After the procedure, the patient must intensively rinse their mouth with 0.2% chlorhexidine for 14 days. 

**Figure 6:**
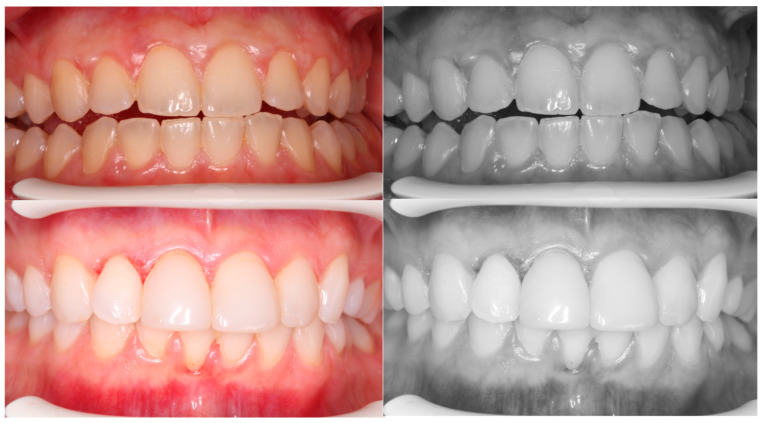
The new zenith of the gingiva and bone must coincide with the zenith of the teeth.

The stitches are removed after 2 weeks. Patients are not allowed to brush their teeth for 21 days after the procedure. After three weeks, the patient begins cleaning their teeth with a brush with very soft bristles. After CCL surgery, the final restoration can be performed after 3 to 6 months (Figure 6).

## 3. Results

A description of the methodology for diagnosis and treatment of patients with abnormal biological width in the case of passive tooth eruption in a patient without composite restorations (Figure 1A and Figure 3B,D) and with composite veneers (Figure 1B and Figure 3C,E) was presented. A satisfactory aesthetic and a new zenith of the gingiva were obtained (Figure 6). It is very important that the gingiva and bone zeniths have a uniform outline and coincide (see Figure 5C,D). 

**Figure 7:**
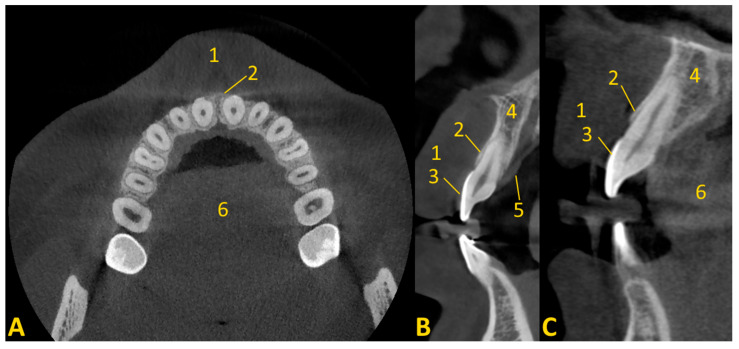
Cone beam computed tomography without soft tissue maneuver (invisible outline of the marginal gingiva from the vestibule side). Cases of soft tissue retraction, after viewing the CBCT images, can be assessed to clearly distinguish between the gingiva and the soft tissue on the palate. In cases where soft tissue retraction is not available, we are unable to clearly differentiate between the lip and gingiva: (**A**) axial view; (**B**,**C**) cross-sectional view. 1—lip; 2—bone edge margin; 3—enamel; 4—alveolar bone; 5—soft tissue of palate; 6—tongue.

This study on the soft tissue retraction maneuver in CBCT aimed to assess the efficacy of a simple imaging technique that enhances the diagnostic precision of both soft and hard tissues preceding CCL surgeries. Enhanced visualization of periodontal structures was noted as a significant benefit of employing the soft tissue retraction maneuver during CBCT examinations. It is obtained through a more distinct demarcation of the cemento-enamel junction relative to the alveolar bone margin. Such enhanced clarity facilitates a more accurate determination of the necessary extent of gingival and bone resection required for CCL procedures. This improvement is particularly notable upon comparison of CBCT images acquired with and without the implementation of the maneuver. A significant enhancement in the delineation of crucial anatomical landmarks was observed. Without the application of a retraction maneuver, the periodontal soft tissues frequently amalgamate within the imaging with other soft tissues, such as the patient’s lips or cheeks, complicating the precise evaluation of the patient’s gingival height and thickness (Figure 7).

Furthermore, the findings underscore the utility of CBCT coupled with a soft tissue retraction maneuver in the precise assessment of biological width, a measure previously challenged by the limitations of two-dimensional imaging techniques and invasive probing methods. This approach significantly complements traditional methods, offering a non-invasive, accurate measurement of the histological sulcus, junctional epithelium, and connective tissue attachment, aligning closely with the established mean distances reported in the literature.

## 4. Discussion

This study provides a detailed examination of the substantial progress in diagnostic imaging for CCL surgeries, attributed to the incorporation of the soft tissue retraction maneuver into CBCT protocols. The deployment of this technique significantly amplifies the visualization capabilities of periodontal structures, thereby refining the accuracy in both the planning and execution phases of CCL surgeries. The insights garnered from this research offer a profound understanding of how technological advancements intersect with clinical practices in the sphere of aesthetic dentistry, potentially setting benchmarks for future methodologies. The application of aesthetic principles such as the golden ratio by pioneers like Lombardi and Levin, alongside the introduction of the recurring aesthetic dental assessment by Ward, underscores a methodical approach to dental aesthetics. These frameworks provide a scientific underpinning for smile design, echoing the ancient pursuit of aesthetic excellence while catering to individual patient preferences and anatomical considerations [1,2,3,4,5].

Biological width comprises the distance between the bottom of the histological gingival sulcus and the alveolar bone [33,34,35,36,37,38]. According to a study by Gargiullo et al. [39], mean distances for biological width are histological sulcus (0.69 mm), junctional epithelium (0.97 mm), and connective tissue attachment (1.07 mm). The total biological width is 2.73 mm. Exceeding the biological width causes chronic inflammation around the crowns, bleeding and overgrowth of the gingiva, unfavorable aesthetics, loss of connective tissue attachment, or bone resorption. Unmaintained biological width may occur in incorrectly designed dental fillings or crowns and prosthetic onlays [6,32,36,37,38]. It is assumed that a 3 mm distance protects against periodontal attachment loss and other adverse effects [11]. In recent studies, the term ‘biological width’ has been replaced by the term ‘supra-alveolar attachment’. This attachment refers to the sum of the epithelial and connective tissue attachment [40]. 

The basic methods of assessing the depth of the gingival pocket and determining the preservation of the biological width include a clinical examination with a periodontal probe (measuring the depth of the periodontal pocket, gingiva puncture) and bite-wing radiographs providing a visual representation of the height of the alveolar ridge and the relationship between the tooth, bone, and restorative margins. These radiographs are invaluable in assessing the bone level, detecting any bone loss, and evaluating the adequacy of the space between the restorative margin and the bone [32].

The routine use of probe measurements and incisions to gauge periodontal health introduces potential variability in clinical outcomes. The probes, typically marked at 1 mm intervals, lack the precision necessary for more nuanced assessments, contributing to the variability [40,41]. Additionally, the technique of creating punctures with endodontic hand tools for measurement purposes, followed by quantification using electronic calipers, not only is invasive but also lacks repeatability. In addition, it requires anesthesia. This method’s invasiveness raises concerns about patient comfort and increases the risk of potential tissue damage, while its subjective nature may compromise the reliability of diagnostic conclusions. In addressing the limitations of current diagnostic methods in dentistry, it is crucial to emphasize the advancements in imaging technologies, particularly the capabilities of cone beam computed tomography. CBCT stands out for its ability to provide objective and highly precise measurements of dental and maxillofacial structures, offering a measurement scale with an exceptional resolution. This level of precision significantly surpasses that of traditional radiographic methods and manual probing techniques, facilitating a more accurate assessment of anatomical features, pathological lesions, and the spatial relationships between different oral structures [42]. The integration of the soft tissue retraction maneuver into CBCT analyses marks a critical transition toward more nuanced and comprehensive imaging of periodontal tissues, encompassing both the soft and hard tissue spectra. The precision in demarcating the cemento-enamel junction from the adjacent alveolar bone margin emerges as a crucial factor for dental practitioners striving to achieve exemplary aesthetic results in CCL interventions. Such enhanced diagnostic clarity not only facilitates meticulous surgical planning but also diminishes the likelihood of postoperative complications by safeguarding the integrity of the biological width. This study accentuates the transformative potential of CBCT when augmented with soft tissue retraction, suggesting a paradigm shift in preoperative diagnostic evaluations.

We encourage the specialists in the field of dentistry to implement the soft tissue retraction every time a CBCT examination is performed, even for other reasons (endodontic, implantological, periodontal, surgical, or orthodontic reasons). Tissue retraction also enables the assessment of the periodontium, the presence of bone dehiscence and fenestration, and the planning of a procedure to cover the recession. It is possible to assess how thick the soft tissue of the palate and maxillary tuberosity is. Without invasive methods, it is possible to know before the procedure how much free gingival graft or connective tissue graft can be obtained. The collected tissues can be used to cover recession or to improve the condition of soft tissues around implants. When retracting the tissues of the palate, it is worth placing gauze on the tongue.

The imaging conditions were 110 kV, 5 mA, voxel size of 0.2 mm, and FOV of 7 × 10 cm. These conditions were adequate according to the literature [43,44]. However, examination with a 0.2 mm voxel size provides an average spatial resolution of 0.4 mm. Therefore, it can distinguish objects with a minimum 0.4 mm distance [45]. Physicians must be aware of underestimation of bone volume measured by means of radiographic examination. On the one hand, it provides perfect sensitivity, but on the other hand, it reduces specificity. The proportion of dehiscence diagnosed based on CBCT imaging was approximately 2.5 times greater than that found on direct examination. The proportion of fenestrations was almost three times greater when diagnosed based on CBCT images compared to the gold standard [46]. 

Intraoral scans may be a supporting tool for CBCT. The quality of the scanners is comparable to traditional impressions. Modern programs enable combining CBCT and IOS. The amount of soft tissue can be measured and the gingival phenotype determined. The simultaneous use of IOS and CBCT allows the creation of surgical templates for the removal of soft tissues (gingivectomy) and hard tissues (ostectomy), which is another step in increasing the precision of the procedure [47,48].

Intraoral ultrasonography (US) provides a quick, non-invasive, comfortable examination of soft tissues. The examination uses an I-shaped, hockey stick, or endocavitary probe. The probes must adhere exactly to the area being tested. Probes with higher-frequency ultrasonic beams (around 20–25 MHz) should be used to assess the periodontium. This results in a higher image resolution of up to approximately 64 μm. US allows for the assessment of the quality and quantity of soft tissues, the border with hard tissues (bone, tooth tissue), enamel, dentine, dentin–pulp junction, CEJ, free and attached gingiva, cortical lamina and spongy bone, and dental implants. The greatest advantage of US is the lack of exposure of the patient to radiological radiation. Therefore, the examination can be repeated and the results compared. However, the US is less available than CBCT in a dentist’s office. The operator’s experience is very important and affects the accuracy of the test [22,48,49,50,51].

The advantages of the soft tissue retraction maneuver in CBCT include ease, speed, non-invasiveness, and greater comfort for the patient, obtaining a lot of important information about soft and hard tissues in three-dimensional images (the quality and quantity of hard tissues, the border with hard tissues (bone, tooth tissue), enamel, dentine, dentin–pulp junction, CEJ, gingiva, cortical lamina and spongy bone, dental restorations, and implants), which allows for accurate treatment planning. Moreover, the scans can be used at any time, also for other purposes, the healing effects can be monitored, and CBCT is more commonly available in dental offices than ultrasonography or multidetector *computed tomography (MD-CT)*. CBCT has a lower radiation dose than MD-CT, higher resolution, and shorter examination duration. In the case of US, the information is not as accurate as in CBCT, the precision of the examination depends on the skill of the operator, and the examination is not as repeatable as CBCT. Increasing the precision of mucosal CBCT assessment can be achieved by combining it with intraoral scans. While the advantages of incorporating the soft tissue retraction maneuver in CBCT are evident, the technique is not without its challenges. The main limitation of this technique is artifacts arising in patients with extensive dental restorations and metal orthodontic appliances, although mitigated to an extent by this advanced imaging technique. Furthermore, the efficacy of this diagnostic enhancement is heavily dependent on patient compliance, emphasizing the critical role of clear communication and thorough patient preparation in the preoperative phase. Another disadvantage is radiation protection. Therefore, the authors encourage performing this maneuver during each CBCT examination to obtain as much information as possible and to have a broad clinical and radiological view. This is in line with the ALARA (as low as reasonably achievable) principle. ALARA is about keeping radiation exposure as far as practical within the dose limits while remaining compatible with the purpose for which the activity is undertaken. Radiation doses in CBCT are low compared to classical medical radiology [52]. 

Future research directions and prospects should focus on making the tissue retraction maneuver a routine diagnostic procedure. Standardized testing should be performed on multiple patients for crown-lengthening procedures, recession coverage, and palatal graft harvesting, e.g., CBCT with US would be justified.

## 5. Conclusions

Compared with conventional radiographic imaging, the soft tissue retraction maneuver in cone beam computed tomography prior to clinical crown-lengthening surgery offers an effective approach to the evaluation and diagnosis of soft and hard tissue. This is due to the detailed planning of the aesthetic CCL procedure. The soft tissues of the cheeks and lips are moved away, which perfectly exposes the marginal periodontium. This provides an assessment of the relationship between the cemento-enamel junction, the bone margin, and the marginal gingiva. Such an approach allows one to plan the size of the procedure and achieve superior aesthetic outcomes in dentistry, contributing to the advancement of aesthetic dentistry through a harmonious blend of art and science. 

## Data Availability

The data presented in this study are available on request from the corresponding author. The data are not publicly available due to privacy restrictions.

## Abbreviations

ALARA	as low as reasonably achievable
CCL	clinical crown lengthening
CBCT	cone beam computed tomography
CEJ	cemento-enamel junction
CT	computed tomography
FOV	field of view
IOS	intraoral scan
MGJ	mucogingival line
US	ultrasonography
